# Social media and sociodemographic characteristics associated with general health in breast cancer patients: a cross-sectional study

**DOI:** 10.11604/pamj.2022.43.95.36791

**Published:** 2022-10-24

**Authors:** Hamid Reza Farpour, Faisal Ahmed, Hamid Nasrollahi, Hossein-Ali Nikbakht, Behnaz Dejman, Pedram Keshavarz, Leila Habibi

**Affiliations:** 1 Shiraz Geriatric Research Center, Department of Physical Medicine and Rehabilitation, Shiraz University of Medical Sciences, Shiraz, Iran,; 2 Bone and Joint Diseases Research Center, Shiraz University of Medical Sciences, Shiraz, Iran,; 3 Department of Urology, School of Medicine, Ibb University of Medical Sciences, Ibb, Yemen,; 4 Department of Radiotherapy and Oncology, School of Medicine, Shiraz University of Medical Sciences, Shiraz, Iran,; 5 Social Determinants of Health Research Center, Health Research Institute, Babol University of Medical Sciences, Babol, Iran,; 6 Student Research Committee, Shiraz University of Medical Sciences, Shiraz, Iran,; 7 Department of Diagnostic and Interventional Radiology, New Hospitals LTD, Tbilisi, Georgia,; 8 Media Management, University of applied sciences and technology, Tehran, Iran

**Keywords:** Social media, breast cancer, general health

## Abstract

**Introduction:**

little is known about social media (SM) use among breast cancer (BC) patients and their general health (GH). This study aimed to evaluate the impact of SM and sociodemographic characteristics associated with GH in BC patients during the treatment phases.

**Methods:**

a retrospective cross-sectional study was conducted on BC patients at Shiraz University from December 2017 to August 2020. Data on clinical, epidemiological, and GH information were collected using a general health questionnaire (GHQ-28). Univariate analysis was performed to determine the impact of SM on GH. Additionally, multivariate logistic regression models [odds ratio (OR)] were performed to identify sociodemographic factors that affect the GH of BC patients.

**Results:**

amongst the 353 individuals with BC, 339 (96%) were female. Their mean age was 48.98 ± 11.57 years. Two hundred and thirty (65.2%) patients used SM; the most frequent SM application was WhatsApp by 209 participants (59.2%). Univariate analysis showed a significant social dysfunction among SM nonusers compared to their users (6.68 ± 2.87 vs. 7.87 ± 3.22) and the difference was statistically significant (p < 0.0001). The use of SM for 3 hours or less was associated with less social dysfunction. However, the difference was not statistically significant (22.80± 12.48 vs. 25.21 ± 10.17, p =0.415). Multivariate logistic regression showed that using SM and working outdoors was positively associated with GH (OR = 0.68, 95% CI =0.29-1.59) and (OR =0.92, 95% CI = 0.54-1.57), respectively. However, female gender and use of chemotherapy were negatively associated with GH (OR = 2.96, 95% CI = 0.74-11.72, and OR =1.47, 95% CI = 0.83-2.57), respectively. Age, marital status, educational level, and disease duration were not statistically associated with GH.

**Conclusion:**

using SM and working outdoors directly and positively impact the behavior of people who have BC, while the female gender and those under chemotherapy were negatively associated with GH in BC patients.

## Introduction

Breast cancer (BC) is one of the most prevalent type of cancer among women and the most important cause of mortality after lung cancer in Western countries [1]. Long-term treatments and high tension had long-time adverse effects on the women´s self-esteem, familial function, marriage role, and quality of life [2,3]. As a result, various aspects of life, such as social relationships or work activities might suffer significantly. General health (GH) status can be viewed as an emotional outburst presenting depression, anxiety, or adjustment difficulties. Initial general health in BC women has been identified as an essential predictor of distress throughout the follow-up [4,5]. As a result, identifying the factors associated with better GH at diagnosis may contribute to identifying women at risk for future psychological problems at an early stage [2,3]. Social media (SM) have been powerful communication instruments with high usage rates in most countries worldwide in recent years [6,7]. SM platforms, including mobile technologies and social networking sites, are increasingly used in BC prevention and treatment efforts. Importantly, SM allows the users to generate, share, and receive information through bi- and multi-directional exchanges, which may transcend geographical borders and provide an opportunity for anonymity [8]. Many studies have found that the GH of BC survivors is related to their age, cancer stage, education level, relationship status, salary, and time since the diagnosis of cancer [4,5]. There have been few studies conducted in Iran on GH and related factors in patients with BC [1,3]. This study aimed to investigate the impact of BC and SM on general health status during the treatment phases of the disease and determine the contributing factors.

## Methods

**Study design and settings:** in this cross-sectional study, 353 BC patients who referred to the Imam Reza Clinic affiliated with Shiraz University of Medical Sciences (SUMS) from December 2017 to August 2020 were asked to complete the data collection forms. This study was conducted according to the guidelines approved by the Faculty of Medicine of SUMS and the Medical Ethics Committee (NO: IR.sums.med.rec.1397.140). All patients provided their written consent and filled out the forms willingly.

**Sample size:** based on previous research and the primary goal of this study, r = 0.2 [Association between the time of using SM and general health], the level of confidence, reading power of 95%, and using G Power software version 3 with the two-way assumption, 314 samples were needed, but considering 10% drop in the study, finally, 350 participants were considered as the final sample size in this study [9-11].

**Inclusion criteria:** BC patients with the following criteria were included: 1) Use of the Internet and at least one SM network (Facebook, Twitter, blogs, forums, text messages, Telegrams, WhatsApp, YouTube, Club, Instagram, and Viber). 2) Willingness and ability to respond to the questionnaire. 3) Age: 18 years and older. 4) With diagnosis and/or under treatment for BC at hospitals related to Shiraz University.

**Exclusion criteria:** the loss of the ability to use the Internet and the inability to cooperate were the exclusion criteria.

### Study measures

The data collection contained four parts; the first part was related to demographic information, such as age, number, gender, educational level, and time elapsed after the diagnosis. The second part consisted of the history of their BC, including the duration and use of chemotherapy. The third part of the questionnaire was about SM usage. Also, the fourth part was the General Health Questionnaire 28 (GHQ-28). The Goldberg institute developed GHQ in 1978. GHQ is a reliable questionnaire used widely in public; it has been translated into 38 languages in different cultures [5]. This questionnaire has been validated to evaluate the mental health by Golberg institution for screening in early treatment level, which contained ten psychological aspects. The 28-question form is the only version that includes the criteria in detail for mental pathology. Through factor analysis, the questionnaire is divided into four subscales: Somatic symptoms and the general health status which include items one to seven, anxiety or insomnia which includes items eight to fourteen, social dysfunction which includes items fifteen to twenty-one, and depression which includes items twenty-two to twenty-eight [12]. Low total scores indicate good mental health, and high total scores mean the opposite. The Likert scale was used to score the questions. The score for the “Never,” “Rarely,” “Sometimes,” and “Frequently” options were 0, 1, 2, and 3, respectively. The minimum possible score was 0, and the maximum score was 90. The scores 14 to 21 show the extremity of the situation in each criterion (among the four of them) [13].

### Statistical analysis

The mean ± SD, median, and Inter-Quartile Range (IQR) described the quantitative variables, and frequency (percent) was used for qualitative variables. A one-way ANOVA test was used to compare the means of variables with more than two categories of qualitative ones. Additionally, logistic regression analysis was used to examine separately (raw effects) and simultaneously (adjusted effects) the predictor variables of SM and the main source of using SM (independent variables) with GHQ (dependent variable) and to show their relationship with each other. Since demographic characteristics and other clinical factors could affect this relationship, these variables were considered for adaptation in the multivariate analysis if they had a significance level of less than 0.4 in the univariate analysis. The patients' general health status was divided into two groups with normal general health (Good) and abnormal general health (low or bad), based on the cut-off point specified in the questionnaire instructions and the regression model; having low general health was considered a consequence factor. The odds ratio (OR) and 95% confidence interval (CI) were used to show the effect size in this model. SPSS 20 was used to analyze the data, and P< 0.05 was considered as statistically significant.

## Results

### Demographic characteristics

Table 1 shows the patients' demographic characteristics. Their mean age was 48.98 ± 11.57 years, 339 (96%) were female, and most of them were aged less than 50 years old (51.3%). Most of them were married (79%) and had diplomas (70.5%). The main total GHQ score was 23.84 ± 12.86, and 167 patients (47.3%) were classified as psychologically distressed (low GH). The mean scores for somatic symptoms, anxiety/insomnia, depression, and social dysfunction were 6.96 ± 4.03, 7.14 ± 4.64, 2.62 ± 3.87, and 7.10 ± 3.04, respectively (Table 2).

**Table 1:** demographic characteristics of the patients (n=353)

Variable	Subgroup	Mean ± Standard deviation, N (%)
Age (years)	-	48.98 ± 11.57
Age group	<50	181 (51.3)
	≥50	172 (48.7)
Gender	Men	14 (4.0)
	Women	339 (96.0)
Marital status	Married	279 (79.0)
	Single	74 (21.0)
Occupation	Housewife	235 (66.6)
	work outdoors	118 (33.4)
Educational level	Diploma	249 (70.5)
	University Graduate	82 (23.2)
*Average Duration of Disease (years)	<1	128 (36.3)
	≥1	171 (48.4)
*History of chemotherapy	No	218 (61.8)
	Yes	66 (18.7)

* The inconsistency of frequencies and percentages is due to having missing data.

**Table 2:** assessment of the use of social media and General Health Questionnaire in patients with breast cancer (n=353)

Variable	Subgroup	Mean ± Standard deviation, N (%)
Using social media	No	123 (34.8)
	Yes	230 (65.2)
Main source of use	Mobil	225(63.7)
	Others	128 (36.3)
Types of social media†	Weblog	3(0.8)
	Message	100(28.3)
	Telegram	161(45.6)
	WhatsApp	209 (59.2)
	YouTube	9 (2.5)
	Club	3(0.8)
	Facebook	11(3.1)
	Twitter	1(0.3)
General status of GHQ	Good (normal)	186 (52.7)
	Low (Abnormal)	167 (47.3)
Total GHQ score	-	23.84 ± 12.86
Dimension score GHQ	Somatic symptoms	6.96 ± 4.03
	Anxiety/Insomnia	7.14 ± 4.64
	Depression	2.62 ± 3.87
	Social dysfunction	7.10 ± 3.04

† Some patients may use more than one media, so the number and percentages are greater than the number of samples.

### Relationship between the general health of breast cancer patients and SM

**Figure 1:**
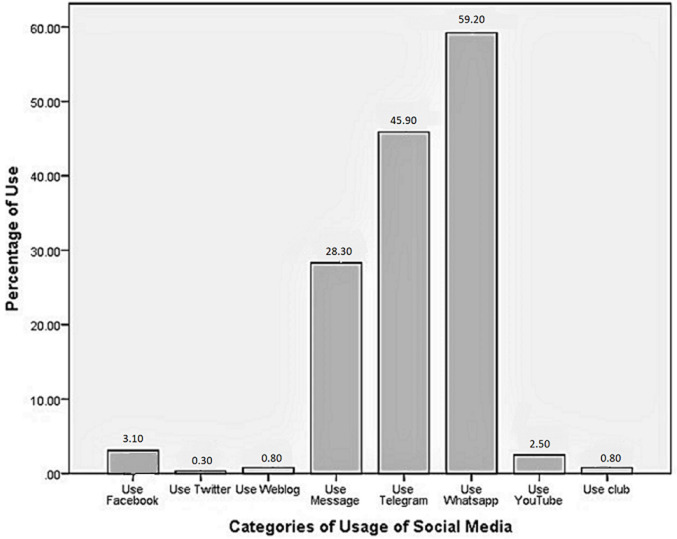
frequency percentage usage of social media types in the study group

**Figure 2:**
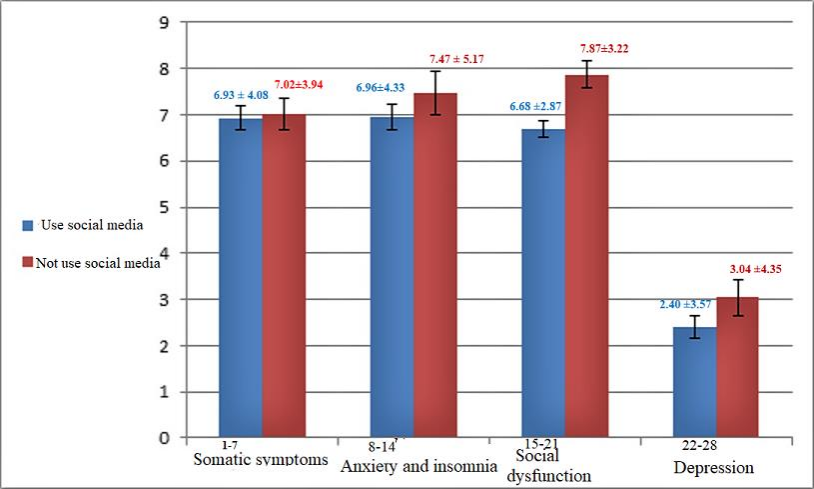
the subgroups of general health status in social media users

Among the 353 BC individuals, 123 (34.8) patients did not use SM, and 230 (65.2) used SM for communication. The most popular application of SM was WhatsApp with 209 persons (59.2%), followed by Telegram application with 161 persons (45.6%) (Figure 1). Generally, our results showed that people who did not use SM in all four scales of GHQ had a higher score and somehow worse GHQ (Figure 2). In comparison between SM users and nonusers, univariate analysis showed a significant social dysfunction among SM nonusers (6.68 ± 2.87 vs. 7.87 ± 3.22), and the difference was statistically significant (p < 0.0001) (Table 3). As to the comparison between using SM for more than three hours vs. less than three hours, there was a general reduction in the mean scale of social dysfunction among SM users for less than three-hour. However, the difference was not statistically significant (22.80± 12.48 vs. 25.21 ± 10.17, p =0.415).

**Table 3:** the relationship between general health status and social media use

Social media status	N (%)	Somatic symptoms	Anxiety and insomnia	Depression	Social dysfunction	Total*
**Not use social media**	123 (34.8)	7.02 ± 3.94	7.47 ± 5.17	3.04 ± 4.35	7.87 ± 3.22	25.42± 13.76
**Use social media**	230 (65.2)	6.93 ± 4.08	6.96 ± 4.33	2.40 ± 3.57	6.68 ± 2.87	23.00 ± 12.31
P-value**		0.850	0.352	0.166	< 0.001	0.092
**less than 3 hours**	211(91.7)	6.82 ± 4.06	6.95 ± 4.40	2.43 ± 3.63	6.58 ± 2.93	22.80± 12.48
**more than 3 hours**	19 (8.3)	8.15 ± 4.24	7.10 ± 3.64	2.10 ± 2.90	7.84 ± 1.77	25.21 ± 10.17
P-value**		0.175	0.887	0.705	0.067	0.415

* Data was presented as (Mean ± SD) **P-values of < 0.05 were considered significant.

### General health-related factors in breast cancer patients

Multivariate logistic regression showed that using SM and working outdoors were positively associated with GH (OR = 0.68, 95% CI =0.29-1.59, and OR =0.92, 95% CI = 0.54-1.57), respectively. While, female gender and using chemotherapy were negatively associated with GH (OR = 2.96, 95% CI = 0.74-11.72, and OR =1.47, 95% CI = 0.83-2.57), respectively (Table 4). Additionally, age, marital status, educational level, and duration of disease were not significantly associated with GH.

**Table 4:** comparison of the patients' general health status and demographic characteristics (n=353)

Variable	Subgroup	Unadjusted		Adjusted	
		*(95% CI) OR	P-value	**(95% CI) OR	P-value***
Using social media	No	Reference group			
	Yes	0.96 (0.61-1.48)	0.856	0.68(0.29-1.59)	0.377
Main source of use	Mobile	Reference group			
	Others	0.92 (0.60-1.43)	0.730	0.61(0.26-1.42)	0.258
Age group	<50	Reference group			
	≥50	0.94 (0.61-1.42)	0.770	-	-
Gender	Men	Reference group			
	Women	3.43 (0.94-12.53)	0.062	2.96(0.74-11.72)	0.121
Marital status	Married	Reference group			
	Single	1.07 (0.64-1.78)	0.795	-	-
Occupation	Housewife	Reference group			
	Work outdoors	0.82 (0.52-1.28)	0.388	0.92(0.54-1.57)	0.779
Educational level	High School	Reference group			
	University Graduate	0.94 (0.57-1.55)	0.820	-	-
Duration of Disease (years)	<1	Reference group			
	≥1	0.91(0.57-1.44)	0.701	-	-
History of chemotherapy	No	Reference group			
	Yes	1.39 (0.80-2.41)	0.243	1.47(0.83-2.57)	0.178

* Cured odds ratio **Fully adjusted odds ratio, adjusted for the main source of use SM, sex, occupation, and chemotherapy use. ***P-values of < 0.05 were considered significant.

## Discussion

This study described the GH-related SM use among BC survivors, compared to nonusers, and examined the sociodemographic characteristics associated with GH. BC is one of the most prevalent cancers among women and the most important cause of mortality after lung cancer in Western countries [1]. The recent data showed an increase in the incidence of BC cancer in Iran [14]. As a result, various aspects of life, such as social relationships or work activities, might suffer significantly [15]. Long-term treatments and high tension had long-time adverse effects on the women´s self-esteem, familial function, marriage role, and quality of life [16]. GH is essential in determining the treatment efficacy in cancer therapy, particularly for cured cancers like BC [5]. GH status can be viewed as an emotional outburst presenting depression, anxiety, or adjustment difficulties. Initial GH in BC women was identified as an important predictor of distress throughout the follow-up [17]. As a result, identifying the factors associated with better GH may contribute to identification of women at risk for future psychological problems at an early stage [2]. In the current study, the patients´ mean age was 48.98 ± 11.57 years, 79% of them were married, 70.5% had lower education, and 18.7% received chemotherapy. Similarly, Fernandez *et al*. investigated the health-related quality of life among 896 BC cases and 890 control Spanish women, and the mean age of BC patients was 50 years; more than 70% were married, most of them had lower education, and 40% were receiving radiation therapy or chemotherapy [2].

The main total GHQ score was 23.84 ± 12.86, and 167 patients (47.3%) were classified as psychologically distressed (low GH). A similar result was mentioned by Fernández *et al*. in this study; 54.4% of the BC patients were psychologically distressed [2]. Regarding the high prevalence of low GH observed in BC cases in our study, even if it can be overestimated, as has been described with GHQ-28 in people with somatic diseases [2,18], the magnitude and importance of including appropriate screening and attention to psychological problems as part of the critical care for BC patients cannot be overstated. Other studies that used the GHQ questionnaire to assess GH in BC women shortly after surgery found an increased prevalence as well [2].

### Relationship between the general health of breast cancer patients and SM

The participants in this study were more likely to use WhatsApp and Telegram, possibly because access to these media is easier for the wider public. Because the research sample consisted of patients who referred to the Shiraz University of Medical Sciences affiliated hospitals of Nemazi and Imam Reza, there was no entry to all BC patients in another city; so, generalizing this outcome should be done with caution. A similar result was reported by Basirat *et al*. [19]. A previous study found that patients who used SM networks for support had higher levels of psychological well-being [20]. Social networks can help people with BC reduce stress and increase their life expectancy by providing psychosocial support and the ability to communicate with others who have the same disease [21]. According to the result of this study, we observed that only the mean score of the social function dimension in SM users was significantly less than nonusers. Hence, it can be concluded that SM users are less anxious and more willing to communicate in their society. Similarly, Sharma *et al*. found that using SM in Saudi students could make them feel more confident and communicative [22]. O´Leary *et al*. recommend that some educational and training programs and more entertaining interventions in SM should be designed for these patients to increase SM and control the time and frequency of SM use, leading to improved GH and quality of life [23].

Abdalqader *et al*., Shensa *et al*., and Habibi *et al*. reported that the time and frequency of SM usage were significantly associated with well-being and led to insomnia and depressive symptoms [24-26]. In line with previous studies, we observed a general reduction in the mean scale of social dysfunction among SM users for less than three hours. However, the difference was not statistically significant (22.80 ± 12.48 vs. 25.21 ± 10.17, p =0.415). On the other hand, if the patients are addicted to SM and spend many hours on this, it causes inactivity and separation from their families and the community. In contrast, Benetoli *et al*. found that using SM in patients with chronic disease or on medication to interact with others was very convenient, leading to improved health knowledge, social activity, and emotional support. This study showed that using SM individuals to communicate with family and friends in more than half of patients revealed that joining SM groups to help BC patients increased their information and decreased their level of anxiety [27]. We found a relationship between using SM and improving GH scores in all aspects compared to non-users. However, the difference was not statistically significant. In the same line, Attai *et al*. found that participation in a BC Twitter TM-supported group increased the patients´ knowledge regarding the disease condition and management and significantly decreased anxiety levels [16]. The Hendrikje Schleife *et al*. study showed that therapy-related factors and the degree of complex decision-making contributed only marginally to the quality of life. However, social support predicts better mental health and quality of life in many domains [28].

### General health-related factors in breast cancer patients

In our study, using SM and working outdoors were positively associated with GH (OR = 0.68, 95% CI =0.29-1.59 and OR =0.92, 95% CI = 0.54-1.57), respectively. However, female gender and using chemotherapy were negatively associated with GH (OR = 2.96, 95% CI = 0.74-11.72, and OR =1.47, 95% CI = 0.83-2.57), respectively. Age, marital status, educational level, and disease duration were not statistically associated with GH. There are controversies in the result of sociodemographic factors that affected GH in most previous research. For example, in Fernández *et al.´s* study, having lower satisfaction with social support, lower education, working with nightshifts, being in more advanced tumor stage, being under chemotherapy, and having children were negatively associated with GH in BC patients [2]. The author explains the association between low GH with lower education and having children, possibly reflecting differences in coping strategies in less educated women and a greater concern in BC patients with children [2]. Chou *et al*. and Fareed *et al*. identified an increasing trend in health-related Internet use among the survivors though the digital divide remains in Internet access [29,30]. While the female gender in this study was negatively associated with GH, Basirat *et al*. found a significant relationship between the SM use and health scores in the female group [19]. It may be due to the lack of awareness of using the SM among females, our people´s culture, and their lack of accessibility in the female group. Another research in Vietnam showed that the relationship status, economic condition, length of therapy, stress emotions, and sleep late behaviors and patterns of BC patients significantly impacted their GH status [11]. The probable reasons for these controversies are differences in the number of subjects, difference in the type of questionnaire, and sampling method.

Limitations of this study include the relatively small sample size impacting the generalizability of our findings and a cross-sectional study design in which causality could not be ascertained. Additionally, this study was based on the general use of social media rather than general health-related users. As a result, a longitudinal study with a larger sample size and community follow-up is needed to better understand the GH status of patients with BC at National Cancer Hospital and other healthcare centers.

## Conclusion

Our findings indicate that SM networks, particularly WhatsApp, are strongly associated with BC patients´ general health and social dysfunction. As a result, SM networks can be regarded as a viable option for alleviating the providing background knowledge among MS patients. Additionally, using SM and working outdoors directly and positively impact the behavior of people with BC, while the female gender and those under chemotherapy were negatively associated with GH in BC patients.

### What is known about this topic

- This study aimed to evaluate the sociodemographic factors that affect the general health status of BC patients during the treatment phases of the disease;
- Since little is known about the social media use among breast cancer (BC) patients and their general health, we evaluated the effect of social media use on the general health status of BC patients.

### What this study adds

- Our findings indicate that SM networks, particularly WhatsApp, are strongly associated with BC patients' general health and social dysfunction;
- Using SM and working outdoors directly and positively impact the behavior of people with BC, while the female gender and those under chemotherapy were negatively associated with GH in BC patients.
